# The plasticity of olfactory learning in culinary students and matched controls

**DOI:** 10.1007/s11682-025-01055-0

**Published:** 2025-10-10

**Authors:** Andreas Steenholt Niklassen, Henrique M. Fernandes, Emil Linnet, Nicoline Brochdorff Therkildsen, Thomas Hummel, Therese Ovesen, Alexander Wieck Fjaeldstad

**Affiliations:** 1 Flavour Institute, Department of Clinical Medicine, Aarhus University, Aarhus, Denmark; 2 Department of Otorhinolaryngology, Head and Neck Surgery, University Clinic for Flavour, Balance and Sleep, Gødstrup Hospital, Hospitalsparken 15, Gødstrup, 7400 Herning, Denmark; 3 Department of Otorhinolaryngology, Smell and Taste Clinic, Technical University Dresden, Dresden, Germany

**Keywords:** Olfactory training, Magnetic resonance imaging, Structural connectivity, Olfaction

## Abstract

**Background:**

Brain plasticity is essential for experts to develop and maintain a high skill level. The aim was to investigate chemosensory sensitivity and central structural connectivity in culinary students naturally training olfactory abilities throughout the first year of education and compare the findings to matched controls.

**Methodology:**

The population included 24 culinary students and 28 controls at the start of their education and 12 months later. The Sniffin’ Sticks olfactory test of olfactory capabilities for threshold, discrimination, and identification were used. Central olfactory plasticity was investigated with magnetic resonance imaging and diffusion tensor imaging to create a structural connectivity matrix of primary and secondary olfactory processing areas for each participant with the seed at the primary olfactory cortex.

**Results:**

For olfactory function, the threshold worsened from 7.23 to 5.42 for controls (P = 0.01); however, Discrimination increased for culinary students from 12.16 to 13.61 (P = 0.03).Compared to controls,culinary students demonstrated stronger connectivity to the gyrus rectus (t = 2.49 p = 0.02) and had a priori stronger connectivity to the caudate nucleus at baseline (t = 2.7147, p = 0.0091), and at follow-up (t = 2.18, P = 0.03).

**Conclusions:**

Culinary students improved their discriminative olfactory abilities during the first year of their education compared to non-culinary students. The culinary students had apriori stronger connectivity to the caudate nucleus than the controls, which remained present at follow-up. Additionally, the culinary students demonstrated stronger connectivity to the gyrus rectus after the first year of their education compared to controls.

## Introduction

Brain plasticity is essential for experts to develop and maintain a high skill level (Filiz et al. 2022). We obtain new skills, develop them, and refine them through training (Filiz et al. 2022). Training induces structural and functional changes in the brain, which is evident in experts with specific skills: musicians with improved hand motor skills (Amunts et al. 1997), athletes with improved visuospatial abilities and coordination (Di et al. 2012), taxi drivers navigating streets (Maguire et al. 2000), and even radiologists with increased efficacy in interpreting human imaging (Filiz et al. 2022; Harley et al. 2009).

Training is not only for experts; clinicians use olfactory training (OT) as a treatment option for patients suffering from olfactory impairment (Pieniak et al. 2022). The original OT consists of training twice daily for approximately 10 s per odor with four odors, and improves olfactory function for patients with olfactory impairment (Hummel et al. 2009).

Knowledge about OT—particularly natural training—is needed for further improving patients' training paradigms. OT promotes functional neural reorganization (Kollndorfer et al. 2015) and changes in olfactory bulb volume (Negoias et al. 2017). Furthermore, structural changes in the brain have been found in olfactory regions after OT (Reichert and Schöpf 2017; Haehner et al. 2022). Using diffusion tensor imaging (DTI), we can explore structural brain connectivity – this has been used to investigate Parkinson’s disease, Alzheimer's disease, attention deficit disorders, and schizophrenia (Park et al. 2018; Seo et al. 2013; Damaraju et al. 2014; Park et al. 2016). However, this technique has not previously been used to investigate olfactory expertise and learning. Combining this with advanced fiber tractography can produce a structural connectivity matrix of the olfactory system – an olfactory fingerprint (Fjaeldstad et al. 2021; Fjaeldstad et al. 2017). In this study, we aim to investigate natural OT with peripheral and central measures of olfaction in novices training to be experts in gastronomy and a matched control group. Using culinary students is an obvious choice due to their use and training of the chemosensory senses to prepare, improve, and create recipes and dishes during their education.

## Material and methods

### Study design

The participants were included at a baseline visit at the beginning of their education in August 2019 and a follow-up 12 months later in August 2020. At both visits, participants were planned to be tested for olfactory function. In addition, they were scanned with magnetic resonance imaging (MRI) and filled in a demographic questionnaire and a questionnaire on the individual significance of olfaction (ISOO) (Croy et al. 2010). The ISOO is validated in Danish in a Danish population (Tchemerinsky Konieczny et al. 2024).

Unfortunately, due to COVID-19 restrictions a systematic error was encountered with the olfactory testing at the 12-month follow-up in August 2020. Due to safety guidelines at the time, the administrators of the test had to wear gloves, which unfortunately had a smell affecting the results of the Sniffin’ Sticks olfactory testing systematically in both groups. Due to this systematic error, this data was faulty, and were not included in the manuscript. For this reason a third visit for testing olfactory function was planned when the restrictions were lifted, which allowed us to collect the correct olfactory data. The participants were tested at the third visit with the Sniffin' Sticks threshold, discrimination, and identification test after the follow-up (median 153 days (IQR 11) later)). All participants except nine showed up for the third visit, of which six were culinary students and three were controls.

### Participants

The participant group originally consisted of 27 culinary students and 28 controls. Three culinary students dropped out, while none of the controls dropped out. All participants were healthy and had no history of chemosensory dysfunction or diseases affecting chemosensory function or processing.

Culinary students were recruited from Culinary Schools in Denmark: Aarhus Tech, Aarhus; Tradium, Randers; College360, Silkeborg; Viden Djurs, Grenaa. Controls were recruited from Aarhus Tech. The controls were from the following educational programs: Woodworking (n = 11), Carpentry (n = 13), and Hairdressing (n = 4). The participants received a compensation fee for participating.

The inclusion criteria: normal olfactory function (assessed with Sniffin' Sticks (Oleszkiewicz et al. 2019)), aged 18–80, starting in the first year of their education.

Exclusion criteria: rhinal disorders, prior nasal-sinus surgery, asthma, pregnancy, neurological and psychiatric disease, smell/taste disorders, and prior formal culinary training.

#### ISOF

The ISOF questionnaire was used (Croy et al. 2010). The applied version has been validated in Danish (Tchemerinsky Konieczny et al. 2024). It consists of questions in three domains: association, application, and consequence. The association scale includes six questions regarding emotions, memories, and evaluations triggered by olfaction. The application scale contains six questions regarding how olfaction is used in daily life. Lastly, the consequence scale contains six questions reflecting the importance of olfaction in decision-making in daily life. All participants filled out the questionnaire.

### Olfaction

The Sniffin' Sticks (Burghart Messtechnik, Germany) is an olfactory test that consists of felt-pen-like devices instead of dye—containing odors (Oleszkiewicz et al. 2019; Niklassen et al. 2017; Rumeau et al. 2016; Fjaeldstad et al. 2015; Hummel et al. 2007; Kobal et al. 2000; Hummel et al. 1997; Kobal et al. 1996). The test is comprised of three sub-tests for testing threshold (T), discrimination (D), and identification (I) abilities. The T, D, and I scores can then be summed to a TDI score of overall olfactory function ranging from 1–48. The Sniffin' Sticks are validated internationally and nationally in Denmark (Niklassen et al. 2017; Hummel et al. 2007). Cut-off scores are as follows: anosmia (≤ 16), hyposmia (≤ 29.8), and normosmia (> 29.8) (Niklassen et al. 2017). Olfactory testing was performed in a well-ventilated and quiet environment. Participants were told not to drink, eat, or brush their teeth one hour before participation (they could drink water).

### Central olfaction

#### Neuroimaging

All participants were examined at the Department of Clinical Medicine, Aarhus University Hospital, Aarhus, Denmark, using a 3 T Prisma MRI (Siemens, Erlangen, Germany) with a 32-channel head coil. Whole-brain T1-weighted scans were acquired, along with Diffusion-weighted images. The 3D T1-weighted images were attained using an MP2RAGE sequence by the following parameters: TR 5000, TE 2.87, field-of-view 240 mm × 256 mm, voxel-size of 0.9 mm × 0.9 mm × 0.9 mm, flip-angle of 4°, in-plane acceleration factor of 2. A full-spin-echo EPI multi-shell dMRI sequence was used to obtain the Diffusion-weighted images. The sequence was encoded with two-phased opposite Directions of anterior to posterior and posterior to anterior. For the sequence, a single gradient table was used with 210 directions. Nine b0 values, and five non-b0 values (700, 1000, 1200, 1500 and 2500 s/mm2) were employed. The non-b0 values had a total number of acquisitions of 15, 21, 30, 60, and 75, respectively. They were all non-linear and evenly distributed. The following parameters were used: a TR of 2850 ms, TE of 71 ms, flip-angle of 90°, and voxel size of 2 mm × 2 mm × 2 mm. Furthermore, a multiband factor of 3 and an in-plane acceleration factor of 2 were used.

To investigate the central changes of olfaction, we employed olfactory fingerprinting, a method developed by Fjaeldstad et al. (Fjaeldstad et al. 2021; Fjaeldstad et al. 2017) and Fernandes et al. (Fernandes et al. 2015).

To create the olfactory fingerprint, an MRI of the brain was performed, including DTI sequences. In addition, we applied probabilistic tractography to the DTI data, and a map of the brain's structural neural networks was created by combining measures of diffusivity, tractography, and estimating crossing fibres, combined with a whole-brain anatomical atlas. The algorithm and the template being used have previously been applied with success by merging findings and templates from both functional and structural studies related to olfaction to recognize an olfactory cortical network and construct a new parcellation of the primary olfactory cortex (Fjaeldstad et al. 2021; Fjaeldstad et al. 2017). Analysis was focused on primary and secondary areas of olfactory processing with the seed at the new parcellation of primary olfactory cortex (which includes the olfactory regions from the Automated Anatomical Labelling (AAL) parcellation (piriform and entorhinal cortex) and the adjacent olfactory part of the amygdala) and to the following 16 olfactory-related regions (Fjaeldstad et al. 2021; Fjaeldstad et al. 2017): Superior frontal gyrus, dorsolateral part; Superior frontal gyrus, orbital part; Superior frontal gyrus, medial part; Superior frontal gyrus, medial orbital part; Gyrus rectus; Insula; Anterior cingulate and paracingulate gyri; Median cingulate and paracingulate gyri; Hippocampus; Parahippocampal gyrus; Amygdala; Caudate nucleus; Lenticular nucleus, putamen; Thalamus; Temporal pole: superior temporal gyrus; Temporal pole: middle temporal gyrus.

### Olfactory fingerprinting

The olfactory fingerprint uses probabilistic tractography to create a connectivity matrix using seed voxels from the AAL template (Tzourio-Mazoyer et al. 2002). First, we compensate for image artifacts, then fit the anatomical atlas to the brain of each participant by using the AAL template and converting to MNI (Montreal Neurological Institute) space with 90 regions in the brain (including the merged olfactory cortex parcellation from Fjaeldstad et al. (Fjaeldstad et al. 2021; Fjaeldstad et al. 2017)), and finally estimating the structural olfactory connectivity between all regions of the brain. For the pipeline application, the FDT toolbox in FSL was used (version 5.0, http://www.fmrib.ox.ac.uk/fsl/, FMRIB, Oxford).

EDDY and TOPUP from the FSL toolbox were used to reduce eddy gradient current and susceptibility-induced artifacts to compensate for image distortions and correct head motion. This was done using two datasets from the diffusion scans and combining the sets from opposite-phase-encoding directions into a separate dataset, creating a bias-field correction map that was sequentially applied to the datasets for optimal signal quality (Andersson et al. 2003; Smith et al. 2004). Then the brain was parcellated into 90 regions of the modified AAL brain atlas, which included the primary olfactory cortex (Tzourio-Mazoyer et al. 2002), and using the FLIRT instrument of the FDT toolbox was linearly co-registered with the MNI-ICBM152 in MNI space to the T1-weighted structural images of the patients, by geometric registration and nearest-neighbor interpolation (Collins et al. 1994). This process resulted in a transformation matrix joined together by the T1 to DTI native space transformation matrix, which granted direct multidimensional normalization of the AAL template to the MNI space of each participant's diffusion native space (Fjaeldstad et al. 2021).

For modeling crossing fibers for all voxels in the brain, a Markov Chain Monte Carlo sampling algorithm was applied to estimate the local probability distribution of fiber direction and other diffusion parameters (Behrens et al. 2003).

To increase the tracking sensitivity of non-dominant fiber populations, we applied automatic voxel-level estimation of multi-fiber directions (Behrens et al. 2007). Then we applied probabilistic tractography at the voxel level and set a sampling number of streamline fibers to 5000 per voxel to estimate the probability connectivity. A binary mask of each participant's brain area was used for defining boundaries. For defining connectivity between the seed (i) and target (j) voxel, we used the proportion of streamlines leaving voxel i and reaching voxel j (Behrens et al. 2007). The connectivity from voxel to voxel was extended into voxel to region. We calculated the connectivity probability from i to j as the number of fibers in the seed region connecting the two regions and Dividing by 5000 (the number of sampling fibers for each voxel in the brain) multiplied by n (the number of voxels in the seed region i). The connectivity probability was estimated from the seed at the primary olfactory cortex to the other 89 regions in the AAL template (adjusted for the olfactory primary cortex parcellation (Fjaeldstad et al. 2021; Fjaeldstad et al. 2017)). The connectivity probability was further normalized by each region's volume in the number of voxels. Due to no Direction of the Diffusion imaging, the connectivity probability was averaged from i to j and j to i to create an averaged undirectional connectivity probability, which was used for the specification of the structural connectivity strength connecting the olfactory cortex and the other brain regions. The process culminated in a 2× 90 weighted matrix for every participant, representing the central olfactory structural connectivity for both the left and right olfactory cortex. All target region connections from the olfactory cortex were included in the matrix when appearing in more than 50 percent of the participants.

### Correlation analysis

To investigate how the structural connectivity of each of the olfactory fingerprint's brain regions correlates to other olfaction measures, we calculated correlations with the Sniffin' sticks scores of threshold, discrimination, identification, combined TDI, and a questionnaire for the individual significance of olfaction. Bonferroni correction was used to avoid type I errors due to multiple comparisons.

### Data collection method

Study data were collected using REDCap electronic data capture tools at Aarhus University (Harris et al. 2009; Harris et al. 2019). The participants filled out the questionnaires directly into the REDcap database, and the researchers filled in testing data and double-checked for correctness.

### Statistics

STATA/IC 16.1 for Mac (StataCorp, TX) was used for statistical analyses. Means, standard deviation, and 95% confidence intervals are detailed when appropriate. Histograms and Q-Q plots were used to assess normality.

Paired t-tests and Wilcoxon signed-rank test were used for comparisons within groups, as appropriate. Students' t-test and Wilcoxon rank-sum were used between groups, as applicable. Pair-wise Pearson correlations were used for the correlation analysis. ANOVA was used to investigate differences between the different educations in the control group. Violin plots were created using the vioplot Stata module (2012), including a distribution density, a marker for the data median, spikes for upper- and lower values, and indications of interquartile range. Alpha was set at.05.

## Results

### Participants

The groups were comparable by age (t = 1.3, p = 0.19), sex (z = −0.47, p = 0.63), and smoking status (z = −0.39, p = 0.70) (Table 1). For olfactory testing at baseline the culinary students had scores of threshold 7.5(95% CI: 6.22;8.77), Discrimination 12.25(95% CI: 11.37;13.13), identification 15.04 (95% CI: 14.6;15.48), combined TDI score 34.79 (95% CI: 33.14;36.44). The controls had scores of threshold 7.15(6.30;8), Discrimination 12.32(95% CI: 11.7;12.94), identification 15 (95% CI: 14.56;15.44), combined TDI score 34.47 (95% CI: 33.29;35.65).

**Table 1:** Descriptive data of the whole study population. HCPW = hours spent cooking per week. ISOO = Individual significance of olfaction. * = Significantly different between baseline and follow-up within groups

	**CULINARY STUDENTS** **BASELINE** **(N = 24) MEAN (95% CI)**	**CULINARY STUDENTS** **FOLLOW-UP** **(N = 24) MEAN (95% CI)**	**P VALUE**	**CONTROLS** **BASELINE** **(N = 28) MEAN (95% CI)**	**CONTROLS** **FOLLOW-UP** **(N = 28) MEAN (95% CI)**	**P VALUE**
AGE	25.6 (23; 28)	26.6 (24; 29)	-	23.9 (23;25)	24.9 (24;26)	-
SEX (MALE/FEMALE)	13/11	13/11	-	17/11	17/11	-
SMOKERS	9	7	-	12	8	-
HCPW	15.66 (11.27;20.07)	36.16 (26.17;46.16)	0.0007*	4.85 (3.92;5.79)	5.21 (4.17;6.26)	0.58
ISOO	34 (31.5;36.5)	33.7 (29.66;37.76)	0.85	35.54 (33.22;37.85)	35.14 (32.64;37.64)	0.72
ASSOCIATION	10.13 (9.10;11.15)	10.63 (9.03; 12.22)	0.42	10.14 (0.2;11.09)	10.32 (9.13;11.51)	0.74
APPLICATION	11.3 (10.4;12.2)	10.46 (9;11.9)	0.21	12 (10.83;13.17)	11.64 (10.57;12.72)	0.50
CONSEQUENCE	12.59 (11.4;13.76)	12.63 (11.14;14.11)	0.94	13.39 (12.58;14.21)	13.18 (12.29;14.07)	0.55

Furthermore, the baseline olfactory testing were comparable between culinary students and controls for scores of: threshold (t = 0.48, p = 0.63)), discrimination (t = −0.14, p = 0.89), identification (t = 0.14, p = 0.89), combined TDI score (t = 0.33, p = 0.74). Culinary students spent more time cooking at both baseline (z = 4.74, p = 0.0001) and follow-up (z = 5.47, p = 0.0001), compared to controls, and also significantly increased time spent cooking within group (z = 3.4, p = 0.0007) (see Table 1). There was no difference between groups or time points for the ISOF scores neither for scores of sub-scales association, application, or consequence nor the total score (Table 1).

At the third control (where only olfactory function were tested – see Table 2), the threshold score remained the same for culinary students (t = 1.86, p = 0.081) but significantly worsened for controls (t = 2.89, p = 0.0081) compared to baseline. Comparing the threshold scores between groups no significant differences were found at baseline (t = 0.48, p = 0.63) or follow-up (t = 0.97, p = 0.34). No significant differences were found between the different education groups (Woodworking, Carpentry, and Hairdressing) for the controls for: TDI: F(2,25) = 0.51, p = 0.6); threshold: F(2,25) = 0.08, p = 0.93); discrimination: F(2,25) = 0.48, p = 0.62); or identification: F(2,25) = 0.91, p = 0.41). The discrimination score increased significantly for culinary students (t = −2.43, p = 0.027) but did not change for controls (t = −1.54, p = 0.14). No significant changes were found for the identification score for culinary students (t = −1.37, p = 0.19) or controls (t = −1.22, p = 0.24) or the TDI score for culinary students (t = −1.07, p = 0.3) or controls (t = 0.98, p = 0.34).

**Table 2:** Descriptive data of the study population excluding participants with no third measurement for the Sniffin' Sticks. T: Threshold. D: Discrimination. I: Identification. TDI: Combination of T + D + I scores. * = Significantly different between baseline and follow-up

	**CULINARY STUDENTS** **BASELINE** **(N = 18) MEAN (95% CI)**	**CULINARY STUDENTS** **FOLLOW-UP** **(N = 18) MEAN (95% CI)**	**P VALUE**	**CONTROLS** **BASELINE** **(N = 25) MEAN (95% CI)**	**CONTROLS** **FOLLOW-UP** **(N = 25) MEAN (95% CI)**	**P VALUE**
AGE	25.56 (22.46; 28.65)	26.5 (23.39; 29.61)	-	23.89 (22.98;24.78)	24.89 (23.98;25.78)	-
SEX (MALE/FEMALE)	10/8	10/8	-	16/9	16/9	-
SMOKERS	6	5	-	10	7	-
T	6.97(5.71;8.23)	5.99(5.10;6.87)	0.08	7.23(6.27;8.18)	5.42(4.61;6.23)*	0.008
D	12.16(11.18;13.15)	13.61(12.64;14.57)*	0.027	12.44(11.77;13.11)	13.08 (12.34;13.81)	0.14
I	15 (14.46;15.54)	15.33 (14.99;15.67)	0.19	15.08 (14.64;15.52)	15.4 (15.02;15.78)	0.24
TDI	34.14 (32.51;35.77)	35.04 (33.33;36.76)	0.25	34.75 (33.5;36)	33.90 (32.53;35.26)	0.29

### Central olfaction

#### Overall olfactory-related connectivity

The overall average olfactory connectivity for culinary students was similar comparing baseline and follow-up (t = 0.46, p = 0.65), as were controls (t = −0.51, p = 0.61).

### Structural connectivity and the left olfactory cortex

Connections to the contralateral olfactory cortex were stronger for culinary students at follow-up than controls (z = 2.05, p = 0.04).

**Fig. 1:**
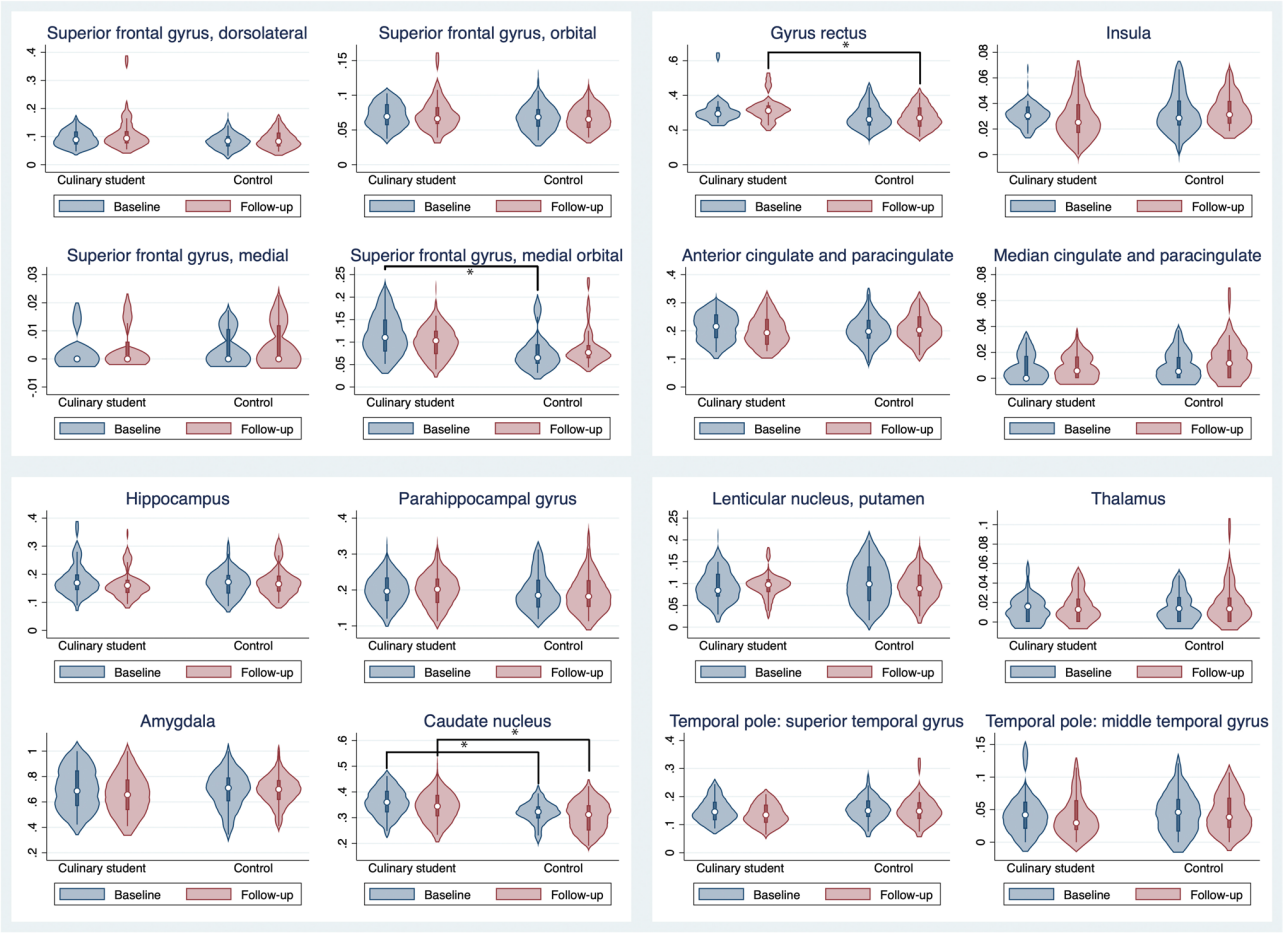
Structural connectivity for the left olfactory cortex. The Violin plots include the median of the data, the interquartile range, and the upper- and lower-adjacent values and also show the values' density. The Y-axis shows averaged, volume-normalized structural connectivity strength between the primary olfactory cortex and the brain regions (see the method section for how this was calculated). The X-axis shows groups. Significant differences are marked with a line and star

At follow-up, the culinary students had a significantly stronger connection to the gyrus rectus compared to controls (t = 2.4853, p = 0.0163), which was not significant at baseline (t = 1.5986, p = 0.1162). A significant difference was found for the caudate nucleus, with stronger connections for culinary students at baseline (t = 2.7147, p = 0.0091) and at follow-up (t = 2.1813,p = 0.0339) compared to controls. There was also a significantly stronger connection to the contralateral caudate nucleus at baseline for culinary students (t = 2.9538, p = 0.0048); however, this was not significant at follow-up (t = 1.2709, p = 0.2096). Culinary students had stronger connectivity at baseline to the superior frontal gyrus medial orbital part compared to controls (t = 2.9919, p = 0.0043), which was not significant at follow-up (t = 1.3158, p = 0.1943). No other connections significantly differed between groups or within groups between baseline and follow-up (Fig. 1).

### Structural connectivity and the right olfactory cortex

**Fig. 2:**
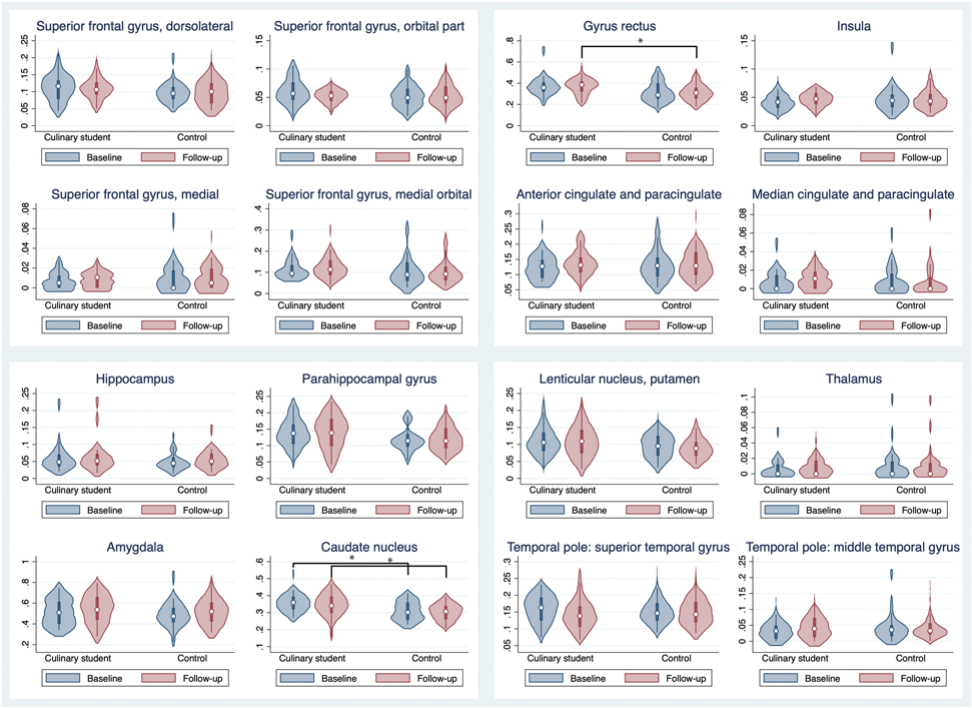
Structural connectivity for the right olfactory cortex. The Violin plots include the median of the data, the interquartile range, and the upper- and lower-adjacent values and also show the values' density. The Y-axis shows averaged, volume-normalized structural connectivity strength between the primary olfactory cortex and the brain regions (see the method section for how this was calculated). The X-axis shows groups. Significant connections are marked with a line and a star

On the right olfactory cortex, the structural connectivity to the gyrus rectus was not significantly different in culinary students compared to controls at baseline; however, there was a significantly increased connectivity in culinary students at follow-up (baseline: t = 1.9710, p = 0.054; follow-up: t = 2.3220, p = 0.0243). Structural connectivity to the caudate nucleus was significantly higher in culinary students than controls at both time points (baseline: t = 3.121, p = 0.003; follow-up: t = 2.2979, p = 0.0258). No other connections significantly differed between groups or within groups between baseline and follow-up (Fig. 2).

### Correlation analysis

At follow-up, the olfactory cortex's structural connectivity and correlation with measures of olfactory function were investigated (Table 3). The correlation was investigated with the ISOO and measures of olfactory function, and no significant correlation was found with any areas of the olfactory fingerprint, when using Bonferroni correction. The closest to significance were the caudate nucleus and putamen at follow up correlated to differences in TDI score. This was further investigated comparing the difference in connectivity between the caudate nucleus at baseline and follow up to differences in TDI scores (0.21, p = 0.13) and differences in putamen at baseline and follow-up were similarily compared to differences in TDI score (0.16, p = 0.26).

**Table 3:** Correlation analysis. The correlation between olfactory fingerprinting connectivity strength was investigated with the Sniffin' Sticks scores of threshold, discrimination, identification, combined TDI score, Individual Significance of Olfaction questionnaire score, and difference in TDI score between timepoints. Bonferroni was used due to the multiple tests, and no correlations were found to be significant. Abbreviations: _1: baseline; _2: follow-up. SF: superior frontal gyrus. Ant. Cing: Anterior cingulate and paracingulate gyri. Median.cing: Median cingulate and paracingulate gyri. TP sup. Temp: Temporal pole: superior temporal gyrus. TP mid. Temp: Temporal pole: middle temporal gyrus

**Olfactory fingerprinting** **targets**	**Olfactory Threshold**		**Olfactory discrimination**		**Olfactory identification**		**Olfactory TDI score**		**Individual olfactory significance**		**Difference in TDI scores**	
	**rho**	**pval**	**rho**	**pval**	**rho**	**pval**	**rho**	**pval**	**rho**	**pval**	**rho**	**pval**
**SFG dorsolateral_1**	-0.054	0.70	0	0.99	-0.001	0.99	-0.055	0.70	-0.102	0.47	0.137	0.33
**SFG orbital_1**	-0.014	0.92	-0.191	0.17	0.177	0.20	-0.053	0.71	-0.085	0.55	0.038	0.79
**SFG medial_1**	0.175	0.22	-0.18	0.20	-0.225	0.11	-0.051	0.72	0.184	0.19	-0.112	0.43
**SFG medial orbital_1**	-0.008	0.95	-0.217	0.12	-0.146	0.30	-0.177	0.21	0.068	0.63	0.101	0.48
**Gyrus_Rectus_1**	-0.024	0.87	-0.167	0.24	-0.074	0.60	-0.145	0.31	-0.113	0.43	0.104	0.46
**Insula_1**	0.068	0.63	-0.148	0.29	-0.052	0.72	-0.051	0.72	-0.166	0.24	-0.047	0.74
**Ant.cing_1**	0.040	0.78	-0.058	0.68	-0.143	0.31	-0.065	0.65	0.012	0.93	0.022	0.88
**Median.cing_1**	-0.030	0.83	-0.005	0.97	-0.153	0.28	-0.098	0.49	-0.106	0.45	-0.053	0.71
**Hippocampus_1**	-0.007	0.96	-0.023	0.87	-0.093	0.51	-0.043	0.76	-0.217	0.12	0.077	0.59
**ParaHippocampal_1**	-0.061	0.67	-0.063	0.66	0.112	0.43	-0.030	0.83	-0.205	0.14	-0.001	0.99
**Amygdala_1**	-0.111	0.44	-0.034	0.81	0.069	0.63	-0.066	0.64	-0.143	0.31	0.044	0.76
**Caudate_1**	0.007	0.96	0.061	0.67	-0.025	0.86	0.014	0.92	-0.126	0.38	0.150	0.29
**Putamen_1**	-0.017	0.90	0.197	0.16	-0.083	0.56	0.071	0.62	-0.028	0.84	0.117	0.41
**Thalamus_1**	-0.125	0.38	0.053	0.71	-0.057	0.69	-0.073	0.61	-0.064	0.65	-0.040	0.78
**TP sup. Temp._1**	-0.252	0.07	0.026	0.86	0.077	0.59	-0.141	0.32	0.003	0.98	0.075	0.60
**TP mid. Temp_1**	0.059	0.68	0.058	0.68	0.146	0.30	0.139	0.33	-0.006	0.97	-0.145	0.30
**SFG dorsolateral_2**	0.125	0.38	0.026	0.86	-0.100	0.48	0.071	0.62	0.056	0.69	0.100	0.48
**SFG orbital_2**	0.139	0.33	0.062	0.66	-0.049	0.73	0.125	0.38	0.015	0.92	-0.087	0.54
**SFG medial_2**	-0.052	0.71	-0.102	0.47	-0.259	0.06	-0.200	0.16	0.018	0.90	-0.06	0.69
**SFG medial orbital_2**	0.084	0.55	-0.085	0.55	-0.225	0.11	-0.083	0.56	0.097	0.49	0.102	0.47
**Gyrus_Rectus_2**	0.054	0.70	0.001	0.99	-0.206	0.14	-0.033	0.82	-0.154	0.28	0.082	0.56
**Insula_2**	-0.138	0.33	-0.124	0.38	-0.148	0.30	-0.237	0.09	-0.061	0.67	-0.235	0.09
**Ant.cing_2**	-0.093	0.51	-0.040	0.78	-0.180	0.20	-0.153	0.28	-0.178	0.21	0.106	0.45
**Median.cing_2**	0.014	0.92	-0.104	0.46	-0.061	0.67	-0.090	0.53	-0.217	0.12	-0.007	0.96
**Hippocampus_2**	0.251	0.07	0.009	0.95	0.092	0.52	0.208	0.14	-0.105	0.46	0.047	0.74
**ParaHippocampal_2**	-0.066	0.64	0.119	0.40	-0.067	0.64	0.023	0.87	-0.047	0.74	-0.003	0.98
**Amygdala_2**	-0.003	0.98	0.066	0.64	0.159	0.26	0.102	0.47	-0.121	0.39	0.041	0.78
**Caudate_2**	0.113	0.42	-0.075	0.60	-0.089	0.53	-0.010	0.94	-0.188	0.18	0.315	0.02
**Putamen_2**	0.101	0.48	0.006	0.97	0.004	0.98	0.074	0.60	-0.095	0.50	0.279	0.04
**Thalamus_2**	0.176	0.21	-0.002	0.99	0.100	0.48	0.152	0.28	-0.013	0.93	-0.007	0.96
**TP sup. Temp._2**	-0.022	0.88	-0.090	0.53	0.179	0.20	-0.022	0.88	0.076	0.59	0.020	0.89
**TP mid. Temp_2**	-0.075	0.60	0.032	0.82	0.158	0.26	0.027	0.85	-0.016	0.91	-0.152	0.28

## Discussion

Compared with controls, culinary students had significantly higher olfactory discrimination and better threshold scores after practical olfactory training through culinary school. This difference was measured despite the preexisting increased time spent on cooking at baseline, which might have diminished the effect of sensory stimulation during the training. Generally, OT has been shown to improve olfactory function (Pieniak et al. 2022). Dalton et al. (Dalton et al. 2002) and Mori et al. (Mori et al. 2015) found improvement in olfactory sensitivity specific to the odors trained; however, they did not find this reflected in the Sniffin' Sticks test. Al Aïn et al. (Al Aïn et al. 2019) found that more complex olfactory tasks than passive exposure to odors improved participants' scores in the identification sub-test of the Sniffin' Sticks. Filiz et al. (Filiz et al. 2022) recently investigated olfactory function in sommelier students and found no improvement in Sniffin' Stick scores; however, they reported structural changes in the brain in increased olfactory bulb volume and changes in cortical thickness with an increase in sommelier students in the right entorhinal cortex, but also found a decrease in the left inferior temporal gyrus, triangular portion of right inferior frontal gyrus, left superior parietal, and superior frontal gyri. Similarly, Pieniak et al. found olfactory function of culinary students was similar to the general population (Pieniak et al. 2023). In another study on wine training, the training group showed increased discrimination scores compared with the control group after training (Wang et al. 2021). Besides being used for people with a normal or heightened sense of smell, OT is a recommended treatment for patients with reduced olfactory function. Several studies have shown an effect of repeated exposure to four or more odors, which improved olfactory function measured with the Sniffin' Sticks, primarily in the discrimination and identification sub-tests. Still, an improvement has also been found in the threshold test (Hummel et al. 2009; Fleiner et al. 2012; Haehner et al. 2013; Geissler et al. 2014; Damm et al. 2014; Altundag et al. 2015; Patel et al. 2017; Langdon et al. 2018; Oleszkiewicz et al. 2022). Filiz et al. (Filiz et al. 2022) argue that the Sniffin' Sticks test was developed to investigate reduced olfactory function and, as such, might have a ceiling effect for people with normal or heightened olfactory function, which the present study could support.

A curious finding was that controls deteriorated in threshold function; however, other studies have previously had the same finding (Negoias et al. 2017). The present worsening in threshold score could possibly be related to the COVID-19 pandemic (where education was put on hold or remote). Notably, this coincided with the pandemic lockdown period in Denmark, where participants remained at home, with fewer social interactions, and fewer varied olfactory experiences. Regular exposure to diverse odorants play a crucial role in maintaining olfactory sensitivity (Dalton 2000; Chen et al. 2024; Chen et al. 2023); thus a monotony of smells could diminish the overall stimulation necessary to preserve normal threshold function. Additionally, habituation and cross-adaptation, physiological processes that modulate olfactory responsiveness based on repeated or less varied odor exposure have previously been shown to negatively affect threshold function (Dalton 2000; Chen et al. 2024; Chen et al. 2023). Consequently, the limited range of olfactory stimuli, combined with prolonged indoor confinement and potential masking practices, might have contributed to a decline in olfactory threshold sensitivity among participants in the control group. This might not have happened to the same degree for culinary students, if they practiced culinary arts at home. Furthermore, they were found to spent significantly more time cooking, and could potentially have done this during the lockdown too. However, suppose the threshold worsening is related to the above, it might also have affected more of the general population, and this finding might be novel and warrants further investigation, as recent research shows that odor deprivation lowers olfactory function (Chen et al. 2024; Chen et al. 2023), and future studies should investigate if acute or chronic shifts in environmental odor exposure may influence olfactory function. Furthermore, The worsening in threshold could also be related to occupational contaminants associated with the choice of education – especially wood dust, which is known to increase nasal congestion (Schlünssen et al. 2002), and rhinitis symptoms are frequent in hairdresser apprentices during the first year of education (Foss-Skiftesvik et al. 2017), however, no difference were found between different education groups in the control group.

For structural connectivity, the culinary students were found to have stronger connections to the caudate nucleus at both time points and demonstrated stronger connections to the gyrus rectus and the contralateral olfactory cortex, however the contralateral connectivity strength decreased from baseline to follow up for connections to the superior frontal gyrus medial orbital part and to the contralateral caudate nucleus.

The brain identifies the stimuli from vision, gustation, olfaction, audition and somatosensation first (tier 1), integrating the stimuli in the amygdala, anterior cingulate cortex, and orbitofrontal cortex (tier 2) assigning affective values, and lastly, decision-making happens (tier 3) in the prefrontal cortex, cingulate cortex, striatum, insula and lateral hypothalamus (Rolls 2023).

The caudate nucleus is part of the corpus striatum in the basal ganglia and is primarily a motor association region; however, it is also involved in several non-motor functions, such as learning, memory, reward, emotion, and motivation (Grahn et al. 2008; 2022). Furthermore, it is involved in decision-making and stimulus–response habit learning (tier 3) (Rolls 2023). The basal ganglia and corpus striatum has been suggested to be involved in the olfactory dysfunction of Parkinson’s disease, and reduced dopamine uptake here to be a potential imaging trace of olfactory dysfunction in these patients (Oh et al. 2018), and as dysfunction in this region can reduce olfactory function, increased connectivity may possibly be linked to increased olfaction. The gyrus rectus is part of the medial orbitofrontal cortex and is involved in assigning affective values to the stimuli (tier 2) (Rolls 2023), and this structural connectivity improved with the training of the culinary students. Furthermore, the connectivity to the contralateral olfactory cortex increased for culinary students compared to controls. This could indicate a stronger interhemispheric transfer and broader network-level integration bilaterally, and could potentially have effects on olfactory function.

Connectivity decreased to the contralateral caudate nucleus and to the superior frontal gyrus medial orbital part for culinary students. The superior frontal gyrus are related to higher cognitive functions as spatial orientation and working memory (Boisgueheneuc et al. 2006), and is part of the core olfactory hedonic processing network (Zou et al. 2016).

The significance of these findings remains unclear. Further investigations are needed to assert if some structural connections are related to increased olfactory function when connectivity strengthens, and if some connections are related to improved olfactory function by a decrease in inhibition of olfactory processing.

The correlation analysis showed no significant correlations with the olfactory testing when using Bonferroni to control, however the difference in TDI scores at follow-up and connectivity strength to putamen and the caudate nucleus was the correlations closest to significant levels. Only subtle, but significant, differences were found in olfactory function, and as such, this may have impacted the correlation analysis, however further research might be needed, e.g. in olfactory training in patients with olfactory impairment, to see if bigger longitudinal differences in olfactory function correlate with the brain regions in the olfactory fingerprint.

## Limitations and strengths

Several limitations should be taken into account when interpreting the study's results. The first limitation is the time from follow-up to measurement of olfactory function with the Sniffin’ Sticks, which unfortunately happened due to COVID-19 restrictions. Furthermore, the COVID-19 situation in general might have affected the olfactory function in general, and due to the time period in Denmark testing for COVID-19 was not used in non-hospitalised patients at the time, so no data were available on COVID-19 in the population. Another limitation is that some measurements might not be able to detect improvement of olfactory function due to ceiling effects, especially in the identification sub-test of the Sniffin’ Sticks.

Compared to studies investigating olfactory function using MRI and DTI, the sample size in the present study is comparable. Filiz et al. (Filiz et al. 2022) used 12 sommelier students and 13 controls. The original study of the olfactory fingerprint had a sample size of 16 (Fjaeldstad et al. 2017), and the validation study had a total sample size of 30 (Fjaeldstad et al. 2021). Esposito et al. (Esposito et al. 2022) investigated brain connectivity and olfactory function after COVID-19 infection, including 27 patients and 18 controls. With the present study having 24 participants and 28 controls, this could be categorized as being on the higher end of the scale regarding sample size. Regardless of the current sample size compared to similar studies, it should be kept in mind when interpreting studies with diffusion MRI and tractography in general that the method generally could have potential limitations, as described by Schilling et al. (Schilling et al. 2019). Furthermore, other methodological considerations should be considered when interpreting the results of DTI. For example, multiple fiber crossing within a voxel may not represent the actual fiber trajectory, as the DTI analysis included correction for two crossing fibers within each voxel; three or more crossing fibers may lead to errors when using a tractography algorithm, which might also cause problems with fiber bundle fusions, divisions, and angulations. However, the current DTI method for assessing the structural olfactory connectivity in the brain has been validated (Fjaeldstad et al. 2021) and represents a solid model for an olfactory fingerprint. The study design with repeated connectivity measures of both culinary students and controls represents a good methodological platform for assessing structural changes over time.

When designing future studies regarding OT in normal and heightened olfactory function, it would be worth considering adding more complex measurements of olfactory function beyond the Sniffin' Sticks, such as identifying components in a mixture (Filiz et al. 2022; Poupon et al. 2018). Furthermore, combining functional and structural connectivity and grey matter changes in future studies of olfactory function, including olfactory bulb volume, might generate further knowledge.

## Conclusion

Culinary students improved their discriminative olfactory abilities during the first year of their education. Furthermore, the culinary students had apriori stronger connectivity to the caudate nucleus than the controls, which remained present at follow-up. Additionally, the culinary students demonstrated stronger connectivity to the gyrus rectus than controls after their first year of education.

## Data Availability

The datasets generated and/or analyzed during the current study are available from the corresponding author on reasonable request.

## Code availability

For the pipeline application, the FDT toolbox in FSL was used (version 5.0, http://www.fmrib.ox.ac.uk/fsl/, FMRIB, Oxford).
